# Facilitating Students’ Creativity, Innovation, and Entrepreneurship in a Telecollaborative Project

**DOI:** 10.3389/fpsyg.2022.887620

**Published:** 2022-04-29

**Authors:** Rustam Shadiev, Suping Yi, Chuanwen Dang, Wayan Sintawati

**Affiliations:** School of Education Science, Nanjing Normal University, Nanjing, China

**Keywords:** 360-degree video technology, creativity, cross-cultural learning activities, entrepreneurship, innovation, telecollaboration platform, virtual panoramic tour, contextual learning

## Abstract

In this study, telecollaborative learning activities were carried out in virtual learning environments created by the 360-degree video technology. We aimed to facilitate students’ creativity, innovation, and entrepreneurship. Two groups of students, a group of junior high school students from China (*n* = 15) and a group of university students from Indonesia (*n* = 10), participated in the study. Students created cultural learning content using the 360-degree video technology which considered to be creative, innovative, and entrepreneurial, shared it with their international partners on the telecollaborative platform and then watched content of their partners to experience virtual panoramic tours. After that, students exchanged their ideas and comments with each other in order to improve content quality. We investigated whether participation in telecollaborative learning activities positively impacts students’ creativity, innovation, and entrepreneurship. The data were collected through questionnaires and interviews. We also analyzed content created by the participants in learning activities. Two main findings were obtained: (1) technology-supported learning activities improved participants’ creativity, innovation, and entrepreneurship and (2) the participants positively perceived their learning experiences. Based on our results, we proposed several suggestions and derived some implications.

## Introduction

To promote social and economic development of society, we need talented people with variety of twenty-first century skills, e.g., cross-cultural communicative competence, creativity, innovation, and entrepreneurship. Twenty-first-century skills development is a target of many educational programs worldwide (Trilling and Fadel, 2009; Core Competencies Research Group, 2016; Huang et al., 2017; Halinen, 2018). These programs consider a learning process to be in a way in which students learn new knowledge and then apply it to solve real-world problems in creative and innovative ways (Lin et al., 2020).

With recent technological advancement, the application of technology in the field of education has dramatically increased, including its usage to promote cross-cultural communicative competence. For example, variety of technologies (e.g., email, Skype, discussion board, social networks, etc.) were used to support communication and information exchange among students from different cultural backgrounds in cross-cultural learning projects (Chen and Yang, 2016; Shadiev and Huang, 2016, 2020; Shadiev et al., 2021a; Shadiev and Dang, 2022). Chen and Yang (2016) explored how Web 2.0 technology (i.e., Wiki platform) and learning management system (i.e., Moodle) can be effective in developing language skills and intercultural communicative competence of participants from different countries. Shadiev et al. (2021a) investigated whether learning activities arranged in virtual reality (VR) environments could facilitate cross-cultural understanding and the trait emotional intelligence of the participants with diverse cultural backgrounds. Participants representing “West and East” in Yang et al. (2014) learned about educational technologies, and various strategies for effective cross-cultural online learning were explored. The results of these above-mentioned studies suggest that student communication and information exchange were important for success of cross-cultural learning projects, and technology effectively supported interaction among participants. Most results were positive, and they demonstrated that cross-cultural competencies of the participants were improved.

Our review of the literature also demonstrated that technology supported cross-cultural learning projects helped the participants learn targeted skills (e.g., linguistic, communication or instructional skills). Therefore, we assume that students’ creativity, innovation, and entrepreneurship can be improved through cross-cultural learning activities too. Firstly, intercultural learning emphasizes the interaction and communication of students from different cultures. Students try to communicate information about local cultures to their foreign partners so that foreign partners can learn about them. We speculate that students’ creativity and innovation can be improved through active interaction and communication. That is, students prepare content regarding their local culture in creative and innovative ways and then interact with each other to exchange local cultural information. Such exchange can help them learn about their partners’ cultures. Cross-cultural learning projects can be useful for students to learn how to introduce their culture better, e.g., use creative and innovative approaches to create content and communicate it. Furthermore, interaction and communication with peers and representatives of different cultures can be useful for ideas exchange and getting inspirational ideas. Secondly, by introducing local culture in details, students may improve their entrepreneurial skills. They may think about how to make foreign partners be interested in their local culture or what kind of information can help them attract foreign peers, e.g., to visit them and try their local products.

Our review of studies on technology-assisted cross-cultural learning demonstrated that most of them focus on improving cross-cultural competencies (e.g., knowledge or skills of discovery and interaction), and development of other skills (e.g., creativity, innovation, and entrepreneurship) received very little attention in the related literature. This study was set to address this gap and to add missing knowledge to the field which can be useful for educators and researchers.

Therefore, the present study designed cross-cultural learning activities based on various themes to facilitate creativity, innovation, and entrepreneurship. Activities were carried out in virtual reality learning environments created by the 360-degree video technology. In our telecollaborative project, students created culture-related learning content such as panoramic tours, and content then was shared with foreign partners and discussed in order to improve it. We investigated whether participation in telecollaborative learning activities supported by technology can facilitate students’ creativity, innovation, and entrepreneurship. The following research questions were addressed in the study: (1) Can learning activities of the study promote students’ creativity? (2) Can learning activities of the study facilitate students’ innovation? (3) Can learning activities of the study develop students’ entrepreneurship? (4) What are students’ perceptions of their learning experiences?

## Literature Review

### Creativity

Scholars believe that creativity is associated with divergent thinking (Shadiev et al., 2022a). Mumford (2003) and Sternberg et al. (2012) suggested that creativity means producing original, valuable, novel, and useful products and things. Creativity refers to using individual information and knowledge to generate new and valuable ideas (Zhang and Zhang, 2018). It is an essential component of individual cognitive processing and the psychological quality necessary for completing creative activities (Lin et al., 2020). Creativity is the comprehensive optimization of complex and multi-factors such as knowledge, intelligence, ability, and excellent personality qualities. The following contents, such as creating new concepts, new theories, updating technology, inventing new equipment, new methods, and creating new works, are the manifestations of creativity.

Many studies on students’ creativity have been carried out up to present. Rahimi and Shute (2021) investigated the effectiveness of an educational game to improve college students’ creativity. Muldner and Burleson (2015) collected data from a creative problem-solving task in a digital environment and then devised computational models to classify students’ creativity automatically. Jang (2009) surveyed how web-based technology could be integrated with real-life to stimulate the creativity of secondary school students, and students’ creativity was facilitated by online interactions and the teacher’s inquiry. The above-mentioned studies indicated that technology was beneficial for cultivating students’ creativity.

### Innovation

Innovation refers to any hypothetical, technological, cultural, commercial, or social relationship that has not existed before under the subjective drive of the individual (Kline and Rosenberg, 2010; Kahn, 2018). According to Marin-Garcia et al. (2016), innovation is the process of coming up with, implementing, and using new ideas. Therefore, innovation emphasizes new things. It is guided by the existing thinking mode to put forward opinions different from conventional ideas. Innovation uses the existing knowledge and materials to change the whole or some parts of things so that they can be updated and developed.

Innovation has received considerable attention in education too. Keinänen et al. (2018) measured students’ innovation competencies in the authentic learning environments through various assessment tools. Erdogan et al. (2013) investigated whether the robotics project can help improve students’ innovation literacy. Moyle (2010) built students’ innovation capabilities through information and communication technologies. Their results showed that researchers and scholars cultivated and developed students’ innovation in different situations. Various teaching approaches were applied to cultivate innovation abilities, and students’ innovation was positively influenced and improved.

### Entrepreneurship

According to Lans et al. (2010), entrepreneurship covers the creation of new businesses, generation of self-employment, and detection of opportunities. Entrepreneurship refers to the pioneering thoughts, concepts, personalities and styles of entrepreneurs. Entrepreneurship is a kind of vitality that can continue innovating and growing (Cunningham and Lischeron, 1991; Stevenson and Jarillo, 2007). Individual entrepreneurship refers to creating a new enterprise by engaging in innovative activities under the guidance of personal strength and personal vision (Liñán and Chen, 2009).

Like creativity and innovation, entrepreneurship also attracted attention from scholars. Some studies investigated the students’ attitude toward entrepreneurship (Peterman and Kennedy, 2003; Veciana et al., 2005). Täks et al. (2014) examined how engineering students experience studying entrepreneurship in a course based on constructivist learning theory and the integrative pedagogy model. Von Graevenitz et al. (2010) surveyed whether entrepreneurship education affects intentions to be entrepreneurial uniformly. These results showed that most studies focused on students’ perceptions, reflections and intentions (Boissin et al., 2009; Keat et al., 2011).

### Studies Related to Creativity, Innovation, and Entrepreneurship

In previous sections, we reviewed studies that focused on one ability only. There are also studies that exist in which scholars covered all abilities at the same time. Edwards-Schachter et al. (2015) explored engineering students’ perceptions about creativity, innovation, and entrepreneurship from two different cultural contexts. They found that most students were creative people and considered that creativity was strongly related to innovation and entrepreneurship. Shu et al. (2020) proposed sustainable-oriented creativity, innovation, and entrepreneurship education framework from an educational perspective. Boysen et al. (2020) explored the impacts of creativity and innovation on students’ learning and teacher’ teaching in entrepreneurship education. They found that creativity, innovation, and entrepreneurship brought challenges among students. Badran (2007) researched possible relations with engineering education for students’ creativity and innovation to enhance their skills. Vaidyanathan (2012) discussed cultivating students’ creativity and innovation through technology in STEM education. Jiang and Sun (2015) established a network platform to carry out activities to develop students’ innovative quality and entrepreneurial ability.

These studies show that researchers have paid attention to creativity, innovation, and entrepreneurship. Nevertheless, we cannot ignore the following: (1) There are only few studies that focus on creativity, innovation, and entrepreneurship as a whole, and mostly, scholars explore these abilities separately; (2) empirical studies mostly focus on students’ attitudes, intentions, and perceptions but less on cultivating students’ creativity, innovation, and entrepreneurship; (3) studies on whether students’ creativity, innovation, and entrepreneurship can be developed with cross-cultural activities as the starting point is particularly scarce. In total, with the gradual deepening of the research on technology-supported cross-cultural activities, it is worthy to explore whether students’ creativity, innovation, and entrepreneurship can be cultivated in cross-cultural activities by designing empirical research with the help of VR based on 360-degree video technology.

### Panoramic Tours Created by 360-Degree Video Technology

Virtual reality technology has been proven to have many educational benefits (Slater and Sanchez-Vives, 2016). It is a technology that creates an artificial environment based on a computer’s 3D model, allowing users to immerse in it and interact with the 3D world. The limitation of such technology is that it does not directly deliver the actual situation to the users, but uses the computer to design a similar situation to the real environment. By contrast, 360-degree VR technology can overcome this shortcoming. The difference between 360-degree VR technology and VR is that the former is generated by real-world footage, while the latter is created by computer software. 360-degree VR technology provides immersive experiences that combine images taken by several cameras or one spherical camera to create a spherical image (Shadiev et al., 2022b). 360-degree VR technology enables users to view content from multiple angles. At the same time, users can choose what content to watch according to their own needs. In addition, it supports the use of head-mounted devices to help users gain a higher level of immersion and experience (Rupp et al., 2019; Shadiev et al., 2022b). Therefore, 360-degree VR technology can provide users with contextual experience and a full range of visual, auditory and kinesthetic experiences to ensure the entire presentation of the real situation (Shadiev et al., 2021b).

Recently, researchers pay attention to panoramic tours created by 360-degree VR technology. Panoramic tours enable users to have panoramic view (i.e., horizontal and vertical). For example, in a panoramic photograph, the user can arbitrarily adjust the height and distance of viewing content. Several studies on 360-degree VR technology applications were carried out. Maach et al. (2018) presented virtual tours based on 360-degree technology to promote tourism and help tourists enter inaccessible areas. Ritter and Chambers (2021) examined response factors such as sense of presence, cognitive image, and affective image developed by 360-degree video technology. Additionally, 360-degree video technology was applied to education. Pham et al. (2018) proposed an innovative educational system for bringing construction field trips to the classroom and providing practical experience and safety knowledge for students. Xiao (2000) investigated the potential of using panorama 360-degree VR technology to enhance Web-based library instruction and found that panorama 360-degree VR could be a powerful tool to make a more helpful medium that allowed navigating, viewing, reading, hearing, and remote access to targeted learning content.

Previous studies show that panoramic tours based on 360-degree VR technology can be effectively applied in many fields, such as tourism, health care, and business. At the same time, the usage of this technology in education is also increasing. However, panoramic tours supported by 360-degree VR technology has received little attention in cross-cultural learning projects, specifically those that focused on creativity, innovation, and entrepreneurship. In this study, we plan to guide students to experience the panoramic tours based on 360-degree VR technology in the process of cross-cultural learning and facilitate their skills such as creativity, innovation, and entrepreneurship.

### Telecollaboration

Telecollaboration is also called as virtual exchange. According to Byram (2021), telecollaboration is “generally understood to be an Internet-based intercultural exchange between people of different cultural/national backgrounds, set up in an institutional context with the aim of developing both language skills and intercultural communicative competence through structured tasks.” Several studies were carried out using telecollaboration approach. Ware and O’Dowd (2008) explored the impact of peer feedback on language development in telecollaboration project. Schenker (2012) strove to reveal the college students’ cultural understanding, interest in cultural learning through telecollaboration. Up to now, telecollaboration has been accepted by many scholars. It was identified as a positive trend as it is helpful to teachers’ and students’ development (Helm, 2015). In the study by Sadler and Dooly (2016), telecollaboration reflected a notable change in the mindset of the teachers and a more profound sense of responsibility from the students’ learning. O’Dowd (2013) reported that telecollaboration was a potential tool for supporting cross-cultural development. Firstly, it provided learners with a different type of knowledge as electronic resources when learning about culture. Compared with traditional resources (e.g., textbooks), electronic resources were easier to access for learners. Secondly, telecollaboration could also contribute to the development of critical cultural awareness as learners have opportunities in online interaction to engage in discussion of meaning. Additionally, Belz and Kinginger (2002) highlighted the usefulness of telecollaboration for making learners aware of cultural differences in communication. The present study examined the cultivation of students’ creativity, innovation, and entrepreneurship based on a telecollaboration during cross-cultural learning activities. Students produced cross-cultural panoramic tours and exchanged them with foreign partners on the telecollaboration platform. We assumed that such activities as creating content, sharing it, and exchanging ideas with foreign partners may help cultivate students’ creativity, innovation, and entrepreneurship. We tested our assumption in the present study.

## Materials and Methods

### Participants

Twenty-five students participated in the study. Ten participants (two females and eight males) were university students from Indonesia, and 15 participants (four females and 11 males) were secondary school students from China. We recruited participants using a convenience sampling method. That is, participants who were “convenient” to the researchers were recruited. One researcher in Indonesia and one researcher in China posted an announcement on online student groups of their university/school. In addition, the information about this study was spread through word-of-mouth advertising in researchers’ university/school. Then, the participants who indicated their interest in participating were recruited on a voluntary basis. The Indonesian students’ age ranged from 19 to 24 years old, and the Chinese students’ age was between 12 and 14 years old. Although the participants from China and Indonesia were of different academic levels, none of them withdrew from the study. All of them had intermediate English as a foreign language skills, and they were able to engage in simple conversations.

The ethical issues and approvals on which the research was carried out and the data were collected were considered in the study. Participants were informed of important details of the study (e.g., its purpose, learning activities, duration, etc.) in the beginning. All participants agreed to participate in the study. The participants had no previous experience in telecollaborative learning projects. Therefore, we thoroughly instructed them about learning activities, how to use technology and basic principles of cross-cultural communication.

Three researchers closely worked with the participants (i.e., two researchers were in China and one researcher was in Indonesia) to ensure their smooth participation. In addition, one secondary school teacher was involved to provide instant help to the participants in China given their relatively young age and intermediate English abilities. The teacher helped students when they could not understand certain information in English or needed assistance with technology. The researchers explained to the participants (in China and Indonesia) all steps of learning activities and guided them on how to effectively exchange information and interact with international partners. In addition, to help participants better understand the concepts of innovation, creativity, and entrepreneurship and then use these three concepts accurately, three researchers also provided guidance in learning activities. For instance, before filling out questionnaires, researchers emphasized these three concepts and gave some related examples to help participants understand them better. Before creating cross-cultural content, the researchers provided the participants with several examples in order to help them incorporate these concepts into their content better. When students were creating content, the researchers gave feedback to each participant to help them accurately incorporate innovation, creativity, and entrepreneurship into their cultural work or improve content.

### Research Procedure

**Figure 1:**
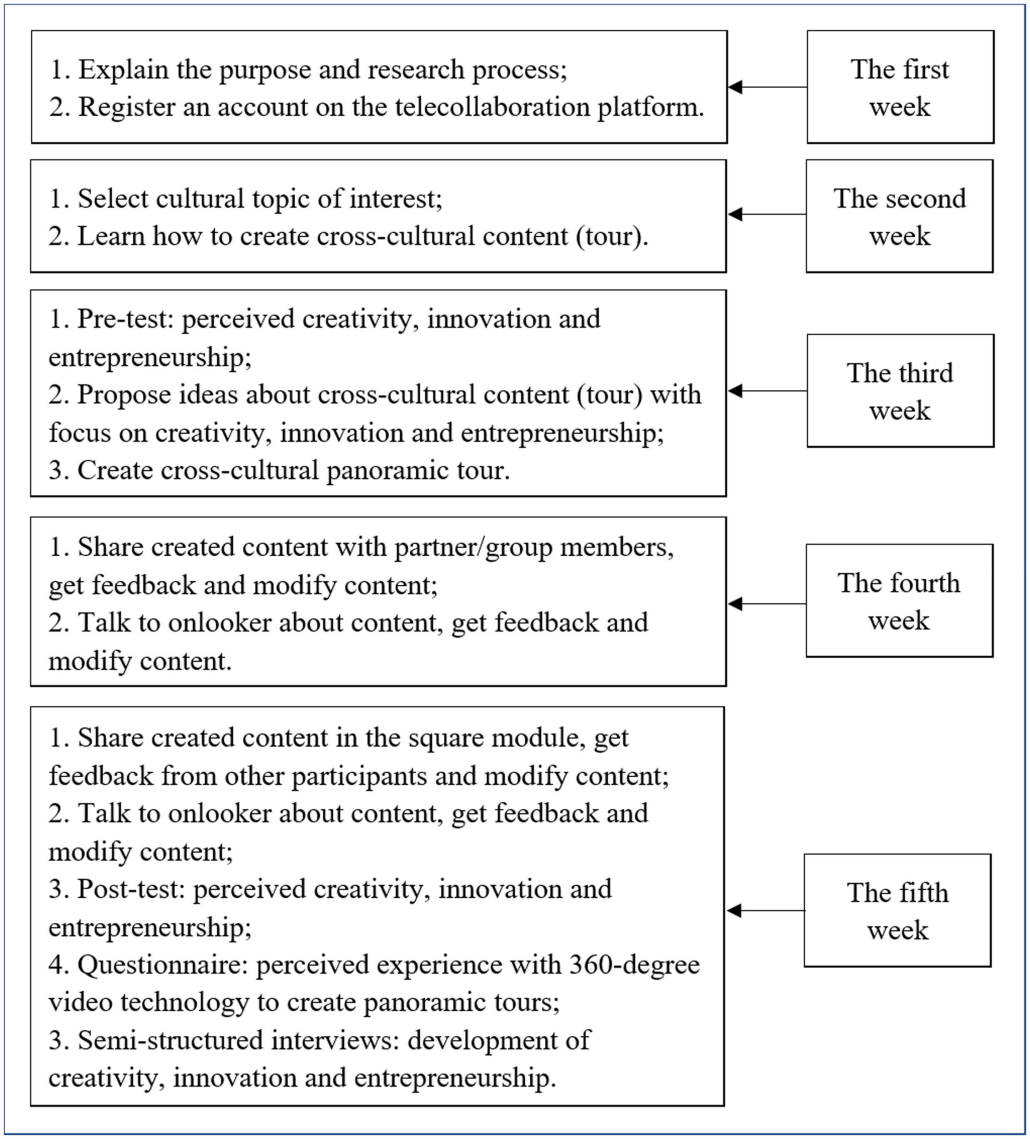
Research procedure.

The research procedure is presented in Figure 1. In the first week, we explained the participants the purpose and learning process of this study. After that, we asked them to register for an account on the online telecollaboration platform developed by the Chinese researchers. In the second week, students were divided into pairs or groups of three (i.e., one student from Indonesia and one or two students from China) according to cultural topics they were interested in, and learned how to create content of panoramic tours focused on creativity, innovation, and entrepreneurship. There were 10 topics, including library, traditional restaurant, classroom, scenic, gymnasium, public square, school playground, school building, community environment and school canteen.

In the third week, students needed to (1) submit their demographic information and their perceived self-efficacy about creativity, innovation, and entrepreneurship—it took about 15 min to provide this information, (2) submit ideas (also called as self-reports) for expected panoramic tours they are going to create—it took about an hour to complete this task, and (3) create cultural panoramic tours—we gave students 5 days to complete their tours. In the fourth week, students needed to (1) upload their created panoramic tours on the telecollaboration platform and then (2) watch and discuss panoramic content shared by partners. The participants communicated with their peers freely using the online telecollaboration platform. After that, students modified their content based on suggestions of their partners. In addition, the participants were asked to show their revised content of panoramic tours to onlookers (i.e., someone who was related to content created by a student—this could be a library manager or staff if content was about a library) to get their feedback and then further improve it. In the fifth week, students uploaded their content to the square module of the telecollaboration platform and discussed it with other students in the square. The square module is a different concept from pair or group because pair or group includes only two or three students but there are more students in the square module such as classmates and foreign partners. After that, students modified their works for the third time according to suggestions of their peers. In addition, final content of panoramic tours was demonstrated to onlookers again to get their concluding remarks. At the end of the fifth week, students were asked to complete a post-test questionnaire on perceived creativity, innovation, and entrepreneurship. In addition, we carried out semi-structured interviews with students to learn about their experiences in the telecollaboration project. Finally, students’ content of panoramic tours was evaluated.

### Data Collection

To address four research questions, both quantitative and qualitative data were collected: the questionnaire of creativity, innovation, and entrepreneurship, proposed ideas, panoramic tour, and one-on-one semi-structured interviews were used to answer the research questions 1, 2, and 3; the questionnaire of perceived experiences in telecollaborative project was used to answer the research question 4.

#### One-on-One Semi-structured Interviews

We interviewed students from China in Chinese and students from Indonesia in Indonesian. Through interviews, we learned about the changes in students’ perceived creativity, innovation, and entrepreneurship in the process of creating panoramic content. Some interview open-ended questions were as follows: (1) Do you think the learning activities were beneficial to develop your creativity? (2) Do you think the learning activities have improved your innovation? (3) Do you think the learning activities were useful in facilitating your entrepreneurship? Each interview lasted approximately 30 min. All interviews were audio-recorded and then transcribed. To analyze content of interviews, two researchers used open coding approach (Strauss and Corbin, 1998). The researchers highlighted and coded those text segments that met the criteria for providing the best research information. After that, the researchers sorted codes to form categories and codes with similar meanings were aggregated together. Established categories of codes then produced a framework to report findings to the research questions. The researchers coded content independently and then compared. If there were significant differences in coding, the researchers resolved them by discussion until a consensus was reached. Inter-rater reliability was over 90%.

#### Perceived Creativity, Innovation, and Entrepreneurship

We measured students’ creativity by the creativity questionnaire from the study of Entrialgo and Iglesias (2020). The creativity questionnaire included six items (see Table 1) and they could be answered on a six-point Likert scale. We designed the endpoints of “strongly disagree” (1) and “strongly agree” (6).

**Table 1:** Perceived creativity before and after learning activities.

#	Items	Questionnaire	Mean	SD	*t*	*p*
1	I often come up with creative solutions to problems.	pre-	3.920	1.115	−6.573	0.000
		post-	5.120	0.726		
2	I am good at providing a fresh approach to problems.	pre-	4.080	0.954	−6.039	0.000
		post-	5.200	0.707		
3	I often come up with new and practical ideas.	pre-	3.800	1.118	−4.956	0.000
		post-	4.920	0.640		
4	I often have new and innovative ideas.	pre-	4.120	1.013	−3.977	0.001
		post-	5.080	0.702		
5	I am good at generating creative ideas.	pre-	4.000	1.155	−5.167	0.000
		post-	5.240	0.597		
6	I often promote and champion ideas to others.	pre-	3.760	1.165	−5.023	0.000
		post-	5.000	1.041		
	Total	pre-	3.947	0.904	−7.006	0.000
		post-	5.093	0.563		

Students’ innovation was measured by the questionnaire of innovation from the study of Marín-García et al. (2013). The questionnaire included 12 items (see Table 2) and they could be answered on a six-point Likert scale. We designed the endpoints of “strongly disagree” (1) and “strongly agree” (6).

**Table 2:** Perceived innovation before and after learning activities.

#	Items	Questionnaire	Mean	SD	*t*	*p*
1	I make proposals appropriate to the demands of the task.	pre-	3.720	1.100	−5.733	0.000
		post-	5.080	0.862		
2	I offer ideas that are original in content.	pre-	3.960	1.136	−5.571	0.000
		post-	5.320	0.748		
3	I offer new ways to materialize the ideas.	pre-	3.800	1.080	−5.866	0.000
		post-	5.240	0.831		
4	I critically evaluate the fundaments of contents and actions.	pre-	3.680	1.406	−4.359	0.000
		post-	4.920	1.038		
5	I identify relationships among different components of the task.	pre-	4.040	1.428	−3.273	0.003
		post-	5.040	0.790		
6	I approach the task from different perspectives.	pre-	4.200	1.118	−3.874	0.001
		post-	5.120	0.726		
7	I use resources ingeniously.	pre-	4.040	1.207	−4.389	0.000
		post-	5.320	0.802		
8	I foresee how events will develop.	pre-	4.000	1.291	−3.091	0.005
		post-	4.880	0.833		
9	I show enthusiasm.	pre-	4.680	1.376	−3.029	0.006
		post-	5.560	0.712		
10	I am tenacious.	pre-	4.520	1.046	−4.028	0.000
		post-	5.400	0.707		
11	I take intelligent risks.	pre-	4.120	1.201	−3.172	0.004
		post-	5.080	0.702		
12	I orient the task towards the target.	pre-	4.000	1.258	−4.112	0.000
		post-	5.240	0.831		
	Total	pre-	4.063	0.941	−5.830	0.000
		post-	5.183	0.515		

We measured students’ entrepreneurship by the entrepreneurship questionnaire from the study of Liñán and Chen (2009). The questionnaire included 11 items (see Table 3), and they could be answered with a six-point Likert scale. We designed the endpoints of “strongly disagree” (1) and “strongly agree” (6).

**Table 3:** Perceived entrepreneurship before and after learning activities.

#	Items	Questionnaire	Mean	SD	*t*	*p*
1	I am ready to do anything to be an entrepreneur.	pre-	3.360	1.411	−3.540	0.002
		post-	4.440	1.193		
2	My professional goal is to become an entrepreneur.	pre-	3.280	1.768	−3.361	0.003
		post-	4.240	1.393		
3	I will make every effort to start and run my own firm.	pre-	4.000	1.384	−3.565	0.002
		post-	5.200	0.866		
4	I am determined to create a firm in the future.	pre-	3.840	1.313	−3.919	0.001
		post-	4.600	1.118		
5	I have very seriously thought of starting a firm.	pre-	3.360	1.497	−3.860	0.001
		post-	4.560	1.261		
6	I have the firm intention to start a firm someday.	pre-	3.600	1.472	−2.789	0.010
		post-	4.360	1.381		
7	Being an entrepreneur implies more advantages than disadvantages to me.	pre-	4.040	1.172	−2.722	0.012
		post-	4.720	1.308		
8	A career as entrepreneur is attractive for me.	pre-	3.680	1.574	−1.937	0.065
		post-	4.240	1.589		
9	If I had the opportunity and resources, I would like to start a firm.	pre-	4.160	1.344	−3.894	0.001
		post-	5.040	1.060		
10	Being an entrepreneur would entail great satisfaction for me.	pre-	3.640	1.411	−5.450	0.000
		post-	5.120	0.781		
11	Among various options, I would rather be an entrepreneur.	pre-	3.360	1.578	−2.551	0.018
		post-	4.000	1.472		
	Total	pre-	3.666	1.211	−4.989	0.000
		post-	4.593	0.952		

We also measured the students’ perceived changes about their creativity, innovation, entrepreneurship (see Tables 4–6) using a questionnaire adapted from Barroso-Tanoira (2017). The items could be answered with a five-point Likert scale. We designed the endpoints of “strongly disagree” (1) and “strongly agree” (5).

**Table 4:** Perceived changes in creativity.

#	Items	Mean	SD
1	Panoramic tour activity helped me realize I’m creative.	5.440	0.651
2	Panoramic tour activity helped me use my creativity.	5.640	0.567
3	I’m more creative now than before this activity.	5.400	0.707
	Total	5.493	0.537

**Table 5:** Perceived changes in innovation.

#	Items	Mean	SD
1	Panoramic tour activity helped me realize I’m innovate.	5.560	0.712
2	Panoramic tour activity helped me use my innovation.	5.440	0.712
3	I’m more innovate now than before this activity.	5.280	0.737
	Total	5.427	0.656

**Table 6:** Perceived changes in entrepreneurship.

#	Items	Mean	SD
1	Panoramic tour activity helped me realize I’m entrepreneurial.	5.200	0.817
2	Panoramic tour activity helped me use my entrepreneurship.	5.480	0.586
3	I’m more entrepreneurial now than before this activity.	5.200	0.646
	Total	5.293	0.547

#### Rubric of Creativity, Innovation, and Entrepreneurship

Students proposed their ideas (also called as self-reports) for cultural panoramic tours with focus on creativity, innovation, and entrepreneurship and then created them in this study. We used a rubric to measure students’ creativity, innovation, and entrepreneurship objectively based on their proposed ideas (before this study) and created content of panoramic tours (during this study) to compare and explore changes in these abilities. The rubric was designed based on relevant literature and standards of several entrepreneurial and innovative competitions. For creativity, we referred to the study of Ferrándiz et al. (2017). For innovation and entrepreneurship, we referred to standards of competitions in two fields (Sun, 2019; Zhao et al., 2020). To ensure the scientific validity of the rubric, two experts in the field were invited. They checked the rubric and gave their comments and suggestions, and then we revised the rubric accordingly.

#### The Questionnaire of Students’ Perceived Experiences to Create Panoramic Tours

We investigated students’ perceptions of their experiences to create panoramic tours using a questionnaire adapted from Bhattacherjee (2001). It included three dimensions (see Table 7): continuance intention (three items), satisfaction (four items), and confirmation (five items). The items could be answered with a five-point Likert scale. We designed the endpoints of “strongly disagree” (1) and “strongly agree” (5).

**Table 7:** Perceive learning experience to create panoramic tours using technology.

#	Items	Mean	SD
Continuance intention			
1	I want to continue creating panoramic tours rather than discontinue its use.	4.520	0.653
2	My intentions are to continue creating panoramic tours rather than any alternative means.	4.200	0.764
3	If I could, I would like to continue creating panoramic tours.	4.640	0.638
	Total	4.453	0.552
Satisfaction			
1	I am satisfied with my decision to create panoramic tours.	4.640	0.490
2	My choice to use 360-degree video technology to create panoramic tours was a wise one.	4.400	0.577
3	I am happy with my earlier decision to create panoramic tours.	4.640	0.490
4	My experience with using this technology to create panoramic tours was very satisfactory.	4.600	0.500
	Total	4.570	0.379
Confirmation			
1	The creation of panoramic tours for intercultural learning meets my expectations.	4.680	0.476
2	This 360-degree video technology gives me all the information and tools needed for intercultural learning.	4.200	0.0707
3	My intercultural learning experience *via* creating panoramic tours falls short of my expectations.	4.400	0.764
4	The sense of presence provided by panoramic tours meets my expectations.	4.520	0.653
5	I generally get the level of service I expect from panoramic tours.	4.200	0.866
	Total	4.400	0.526

All instruments used in the present study were adopted from existing instruments: the creativity questionnaire (Biraglia and Kadile, 2017; Entrialgo and Iglesias, 2020), the innovation questionnaire (Marín-García et al., 2013; Watts et al., 2013), the entrepreneurship questionnaire (Liñán and Chen, 2009; Karimi et al., 2012; Entrialgo and Iglesias, 2020), the perceived changes in creativity, innovation, entrepreneurship questionnaire (Barroso-Tanoira, 2017), and the perceived learning experience questionnaire (Bhattacherjee, 2001; Henriksen et al., 2021; Juliana et al., 2021). All of them were widely used in educational research, and scholars proved their validity and reliability. Nevertheless, two professors who are experts in educational science checked items of these questionnaires to ensure that they are valid and can be used for the present research. No issues aroused during the validation and the questionnaires were used after the experts’ confirmation.

## Results

### Creativity

To answer the first research question, we analyzed the results of pre- and post-questionnaire. In addition, we used evaluation of students’ proposed ideas (also called as self-reports) and their created content to see differences in their creativity before and during the study. The paired sample *t*-test was employed to compare the pre- and post-questionnaire results. Table 1 presents the results and according to them, students scored higher on the post-questionnaire (total *M* = 5.093; SD = 0.563) than on the pre- test questionnaire (total *M* = 3.947; SD = 0.904), *t* = −7.006; *p* < 0.000. Table 4 presents results of perceived changes in creativity, and they show that students believed that the learning activities were helpful in improving their creativity (*M* = 5.493; SD = 0.537). Table 8 shows the results of content evaluation (i.e., proposed ideas before the activity vs. created virtual tours), and according to them, students’ scores on creativity are higher for created virtual tours (total *M* = 3.310; SD = 0.761) than for proposed ideas (total *M* = 2.110; SD = 0.495), *t* = −10.733; *p* = 0.000.

**Table 8:** Changes in creativity: evaluation of proposed ideas and created content and their comparison.

#	Items	Variable	Mean	SD	*t*	*p*
1	Fluency	Proposed ideas	2.040	0.539	−10.007	0.000
		Created content	3.800	1.041		
2	Flexibility	Proposed ideas	2.040	0.539	−11.298	0.000
		Created content	3.800	1.041		
3	Originality	Proposed ideas	2.160	0.554	−7.905	0.000
		Created content	3.520	1.046		
4	Elaboration	Proposed ideas	2.200	0.577	−8.777	0.000
		Created content	3.920	1.116		
	Total	Proposed ideas	2.110	0.495	−10.733	0.000
		Created content	3.310	0.761		

In addition, we provide two extracts from interviews with students that evidence how their creativity improved.

#### Student ID1

*This activity has boosted my creativity, and I have more ideas now for presenting my work. The theme of my work is about the classroom, and at the beginning of my self-report (or proposed ideas), I only thought about introducing items like “what is it?” But after discussing and exchanging ideas, it occurred to me that I could describe the items in detail. Thus, in my final work, I introduced a lot of objects from different angles. For example, when I introduced the flag, I thought about these questions, “What is it? Why is it in the classroom? What are the implications of the flag?” Until now, I have a lot of ideas.*

#### Student ID2

*My creativity has increased through this activity. In this project, I used 360 video technology to show a duck blood vermicelli soup restaurant in Nanjing. In my self-report (or proposed ideas), I only briefly introduced the menu and facade decoration. And after the exchange of information, I came up with other ideas about introducing food culture, so I think my creativity has improved.*

Another evidence about improvement of creativity through this project is comparison between student content of proposed ideas (before learning activities) and their final work such as created panoramic tours (after learning activities). Here is the extract from self-report (or proposed ideas) of student ID3:

*My selected theme is classroom. My school, Nanjing Zhonghua Middle School, has a long history so my work aims to show the style of the junior high school classroom and daily life of students. In my work, my idea is to introduce the infrastructures of the classroom, because it can reflect Chinese classroom culture. I want to make foreign friends know more about the study life of Chinese students through my work.*

**Figure 2:**
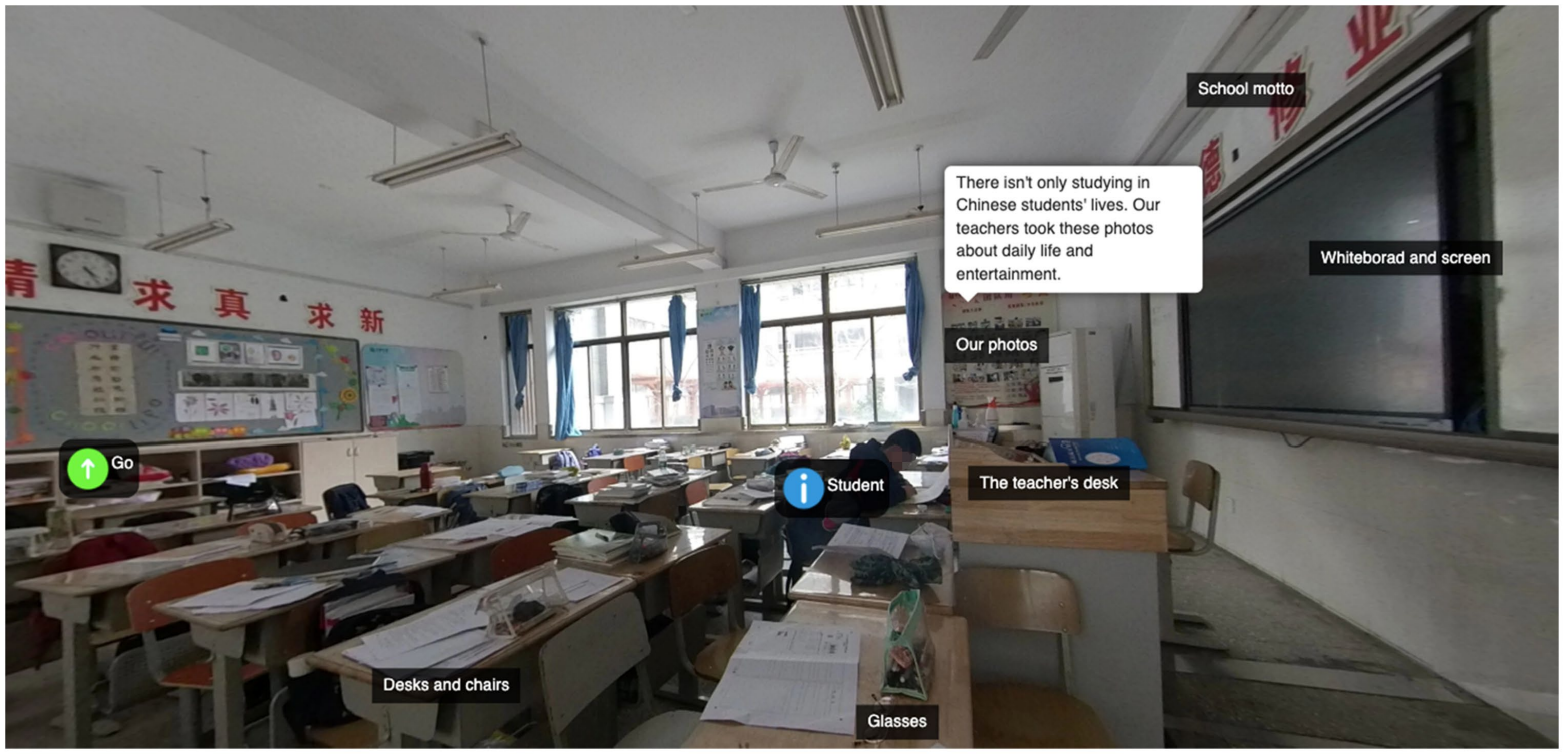
Created content (panoramic tour) of student ID3.

In proposed ideas, student ID3 just had an idea to introduce his work through the classrooms’ infrastructures such as desk, clock, and whiteboard. After communicating with peers and onlookers, he was inspired to introduce some objects in classroom and their characteristic. Final work of student ID3 is captured in Figure 2. We can see in the figure that he explained about the school motto, team corner, national flag, class photographs, students and many other things in his final panoramic tour. So we think that students had more ideas for their final work compared to their ideas they proposed in the beginning of learning activities. All above-mentioned findings (from questionnaire, interviews, and content analysis) suggest that students’ creativity has increased in learning activities.

### Innovation

We answer the second research question through comparing the results of the pre- and post-questionnaire and evaluation of students proposed ideas and created content such as cultural panoramic tours. The paired sample *t*-test was employed to compare pre- and post-questionnaire scores, and the results are reported in Table 2. According to the table, students scored higher on the post-questionnaire (total *M* = 5.183; SD = 0.515) than on the pre- questionnaire (total *M* = 4.063; SD = 0.941), *t* = −5.830; *p* < 0.000. The results of perceived changes in innovation are included in Table 5, and they show that students believed that learning activities were useful to improve participants’ innovation (*M* = 5.427; SD = 0.656). Our evaluation results of proposed ideas and created content and their comparison are reported in Table 9. The results show that scores of created content are higher (total *M* = 3.560; SD = 0.958) compared to scores of proposed ideas (total *M* = 2.080; SD = 0.728), *t* = −9.031; *p* < 0.000.

**Table 9:** Changes in innovation: evaluation of proposed ideas and created content and their comparison.

#	Items	Variable	Mean	SD	*t*	*p*
1	Theme innovation	Proposed ideas	2.480	1.005	−7.905	0.000
		Created content	3.840	1.143		
2	Content innovation	Proposed ideas	1.880	0.726	−7.895	0.000
		Created content	3.400	0.957		
3	Structural innovation	Proposed ideas	2.000	0.646	−9.867	0.000
		Created content	3.520	0.963		
4	Emotional resonance	Proposed ideas	1.960	0.935	−7.268	0.000
		Created content	3.480	0.963		
5	Total	Proposed ideas	2.080	0.728	−9.031	0.000
		Created content	3.560	0.958		

The following are extracts from interviews with students to support our findings about improved innovation.

#### Student ID4

*After comparing the initial self-report with the final work, I think my innovation has improved. In this activity, the theme of my work is the school canteen. In proposed ideas, I only intended to label the canteen’s food. In my final work, I added labels such as sinks, meal card recharge machines, payment machines, and even dining rules, which greatly enriched the details of the work and made it closer to the actual scene I observed. At the same time, I also found that these details are not presented and are different in works of the same theme. So I think my innovation has been boosted.*

#### Student ID5

*This activity has increased my creativity as I label my final work more and introduce more content than my self-reports. My work is about the library, and in the self-report, there were only labels about the book, including the book’s price, the category of the book, the content,* etc. *In my final work, in addition to the above, I also described the store next to the library in the final work. Moreover, I found my work unique compared to works on the same topic, and I provided links to purchase the book, which was not found in other works. Overall, my final panoramic work looks more complete than before.*

Additionally, we tried to get objective evidence to support our claim that participants’ innovation improved in learning activities of the study by comparing content of proposed ideas and final panoramic tours. Here are proposed ideas of student ID6:

*I chose “Gedung Serbaguna Unila” to accomplish my work related to public square. I am going to show the following points: (1) Gedung Serbaguna Unila, including its function, location,* etc.*; (2) because Lampung culture has a uniqueness, so I also show the cultural value of the building, such as carvings, the colors of building,* etc.*; (3) of course, I also will introduce the interior and exterior of the building, including typical Lampung patterns, carvings, history and so on.*

**Figure 3:**
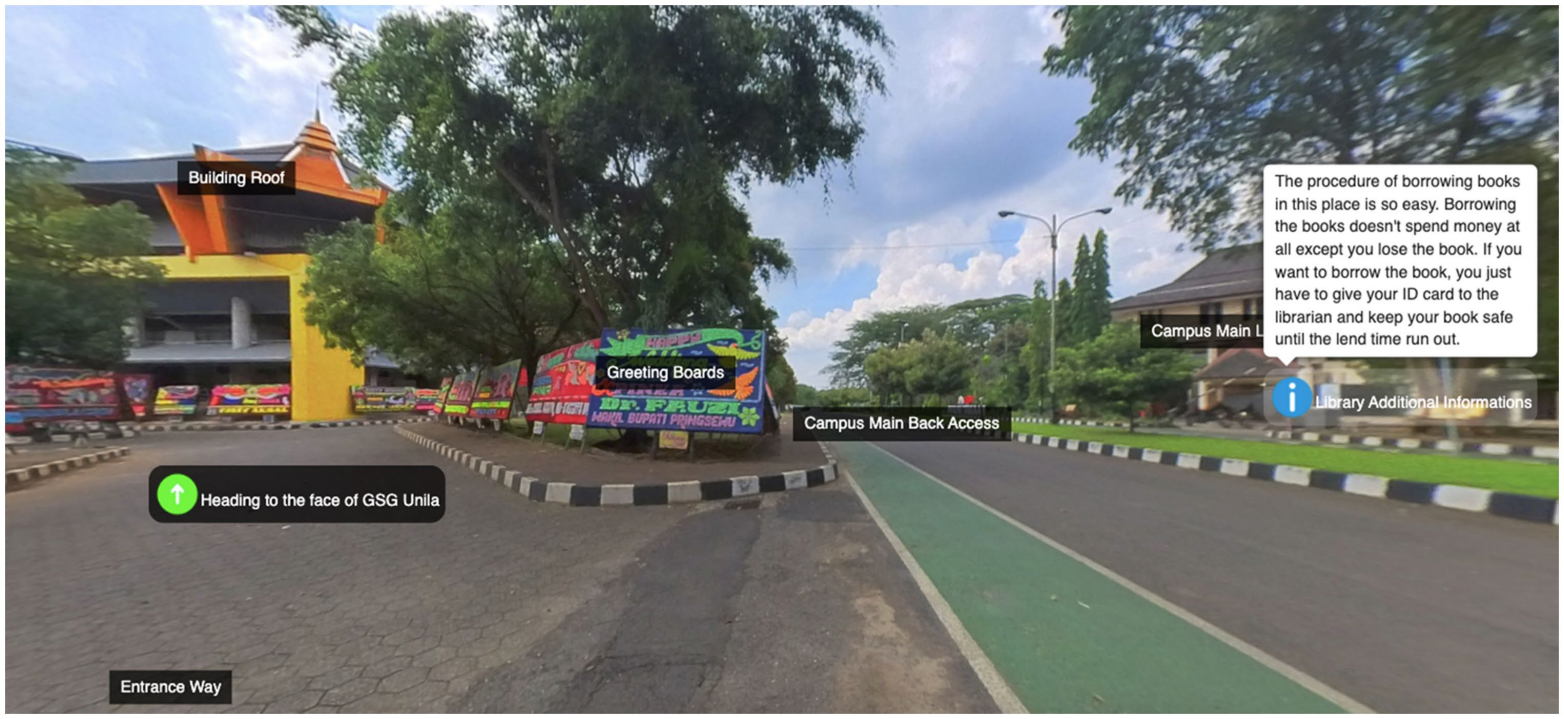
Created content (panoramic tour) of student ID6.

Student ID6 planned to introduce his work through various objects and artifacts, such as carving, parking lot, and building and so he proposed such ideas. After communicating with peers and onlookers, in his final panoramic work (Figure 3), he introduced the library and road next to this place, this is new and different from others’ works. Moreover, he also made a more specific description of the interior and exterior of the building than that at the beginning of the study. This evidence demonstrates that innovative skills of students ID6 improved. Based on the above-mentioned results (from questionnaire, interviews and content analysis), we may conclude that our learning activities were beneficial to improve students’ innovative abilities.

### Entrepreneurship

To address the third research question, we analyzed pre- and post-questionnaire results and evaluation scores of proposed ideas and created content. The paired sample *t*-test was employed to compare the scores of pre- and post-questionnaire, and the results are reported in Table 3. We can see from the results that students scored higher on the post-questionnaire (total *M* = 4.593; SD = 0.952) than on the pre-questionnaire (total *M* = 3.666; SD = 1.211), *t* = −4.989; *p* < 0.000. The results of perceived changes in entrepreneurship are demonstrated in Table 6. According to the results, the participants felt that learning activities were useful in improving their entrepreneurship (total *M* = 5.293; SD = 0.547). In Table 10, scores of proposed ideas and created content are included and compared. The results show that students were scored higher on created content (total *M* = 3.740; SD = 0.959) than on proposed ideas (total *M* = 1.980; SD = 0.494), *t* = −11.018; *p* < 0.000.

**Table 10:** Changes in entrepreneurship: evaluation of proposed ideas and created content and their comparison.

#	Items	Variable	Mean	SD	*t*	*p*
1	Propaganda value	Proposed ideas	2.120	0.666	−8.241	0.000
		Created content	3.760	1.052		
2	Economic value	Proposed ideas	1.880	0.526	−9.655	0.000
		Created content	3.600	1.000		
3	Practical value	Proposed ideas	1.960	0.539	−11.289	0.000
		Created content	3.840	0.987		
4	Prospect value	Proposed ideas	1.960	0.539	−9.859	0.000
		Created content	3.760	1.052		
5	Total	Proposed ideas	1.980	0.494	−11.018	0.000
		Created content	3.740	0.959		

Below are two extracts from interviews with students to support our findings:

#### Student ID7

*The activity improved my entrepreneurship. Now I understand how to promote something for sale. With panoramic tours, one way to let someone know and get their attention, like the Padang cuisine that I introduced in my work. With the help of panoramic photos taken using VR 360, I was able to explain how the food was made and how much it cost and promote the best-selling items. This will make it easier for someone in entrepreneurship. People can also see the menu, place, dining conditions, and location beforehand so they can determine if they what to go there and what they want to try there.*

#### Student ID8

*My entrepreneurship was improved through this activity. This project is an excellent platform for introducing interesting local places and learning about the culture. The 360-degree panoramic tours technology will increase the interest of tourists to visit the site. And if many tourists visit, it will undoubtedly increase the economy of the surrounding community, such as in housing, tour guidance or catering business.*

Comparing content of proposed ideas and final work helped us see the improvement in students’ entrepreneurial abilities. Here are proposed ideas by student ID 9:

*This is the school canteen of the University of Lampung. The special thing about this canteen is that it just serves Indonesian food. There are a lot of food on menu and it is not only for students but also for faculty and workers. Regarding entrepreneurship, I intend to provide menus in this canteen to tell you about the prices of foods and beverages.*

**Figure 4:**
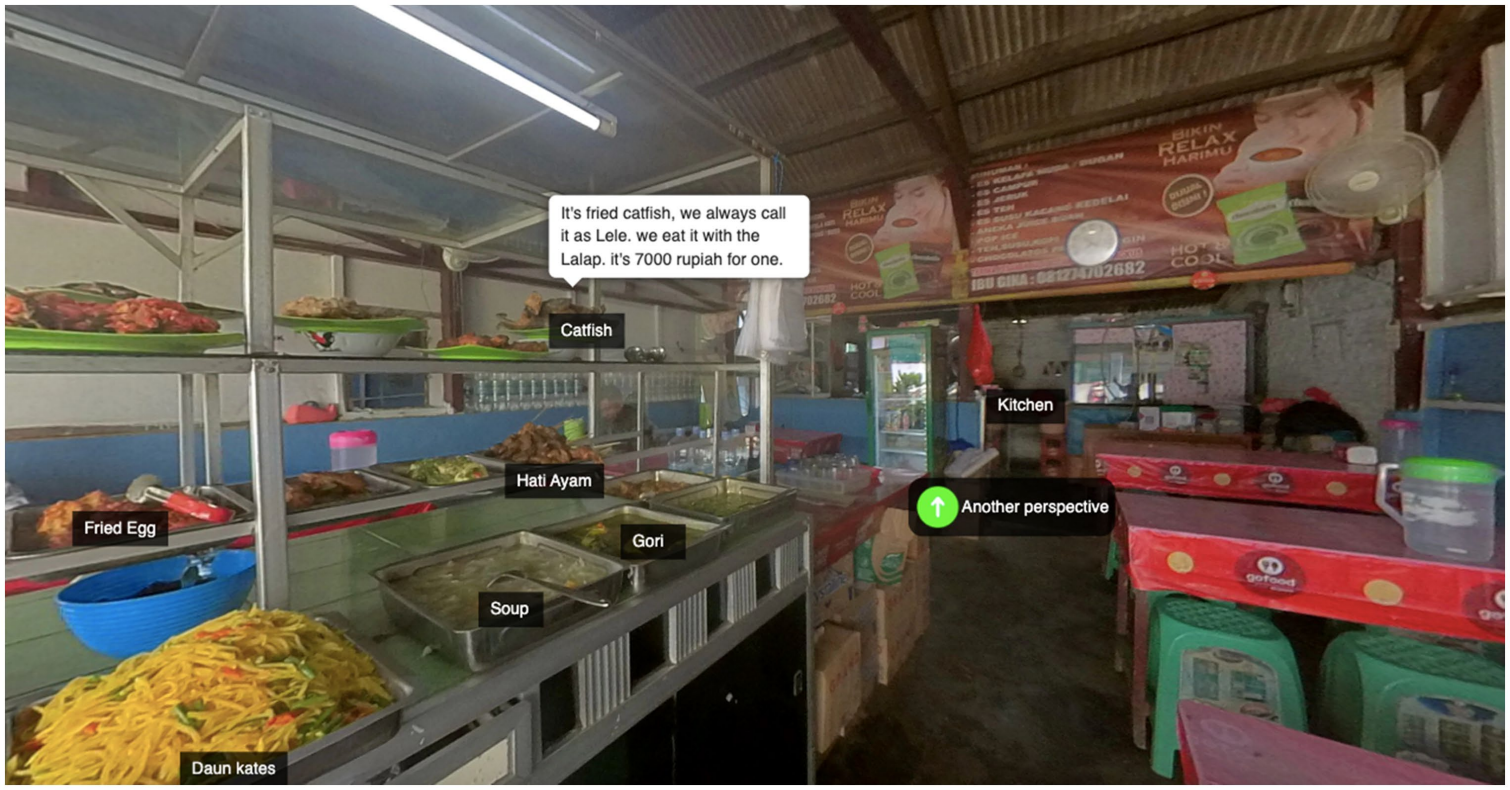
Created content (panoramic tour) of student ID9.

In proposed ideas, student ID9 provided menus of foods and beverages. However, he did not introduce various foods and their prices. After communicating with peers and onlookers, he understood that more information is needed. Final work of student ID9 is captured in Figure 4. Student ID9 introduced foods and their prices on display showcase in detail in his final panoramic work. Moreover, he also introduced various methods of payment. According to the above contents, we concluded that the students’ entrepreneurship was improved. Furthermore, the above-mentioned findings (from questionnaire, interviews, and content analysis) suggest that learning activities were beneficial in enhancing students’ entrepreneurship.

### Perceive Learning Experience

To answer the fourth research question which is related to perceive learning experience to create panoramic tours, we carried out questionnaire survey with the participants. Table 7 reports the results and they show that the participants had positive perceptions of their learning experience. Their level of continuance intention to use technology for creating panoramic tours was high (total *M* = 4.453; SD = 0.552). The participants’ satisfaction to use technology for creating panoramic tours was also high (total *M* = 4.570; SD = 0.379). In addition, the participants confirmed in the questionnaire that using technology for creating panoramic tours met their expectations (total *M* = 4.400; SD = 0.526). The results of the questionnaire showed that the students positively perceived their learning experience using 360-degree video technology to create panoramic tours.

## Discussion

Main purpose of this study was to learn whether our learning activities to create, share and experience panoramic tours using technology can facilitate students’ creativity, innovation, and entrepreneurship. We analyzed students’ responses to questionnaires, evaluated content of proposed ideas and content of panoramic tours, and studied content from interviews. The results showed that technology-supported learning activities helped students improve their creativity. Students had more novel ideas that were reflected in their created panoramic tours. Although few studies involve creativity in cross-cultural learning, this finding aligns with those obtained in previous related studies (Jang, 2009; Zhang et al., 2012; Shadiev and Huang, 2016; Yilmaz and Goktas, 2017; Shadiev and Dang, 2022). The students’ creativity in previous studies were enhanced by various technologies (e.g., smartphones in Shadiev et al. (2022a) or computers in Lin et al. (2020)). The study results of Zhang et al. (2012) showed that visual tools made students more creative. Jang (2009) found that web-based technology stimulated students’ creativity in a science course. In the study of Yilmaz and Goktas (2017), AR was an effective technique to improve students’ creativity in storytelling activities.

In terms of innovation, the results showed that it was enhanced by our technology supported learning activities. That is, after participating in learning activities, students implemented and used more new ideas to create their works. On the one hand, they tag more content than before in their works; on the other hand, they also tag something new and different from others’ works. Our results are consistent with those from previous related studies (Zhang et al., 2012; Erdogan et al., 2013). In the study of Erdogan et al. (2013), scholars found that students’ innovation skills were developed through the robotics programs. Zhang et al. (2012) also found that students’ innovation competence was increased significantly through visual tools.

For entrepreneurship, the results demonstrated that it was improved through our learning activities supported by technology. That is, students’ entrepreneurial intentions and entrepreneurial attitudes were enhanced. Students learned to introduce the price of items for sell to reflect economic value. Moreover, they improved their works’ quality constantly and described their works specifically so as to attract peers and others to go to this place to study, travel, and consume promoted products. This makes students be aware of their entrepreneurial potential. It was also an entrepreneurial manifestation. This result is similar to other related studies about students’ entrepreneurship cultivation after training (Souitaris et al., 2007; Küttim et al., 2014). For example, the entrepreneurship program raised the entrepreneurial intention of science and engineering students in Souitaris et al. (2007).

Another aim of this study was to investigate students’ perceived learning experiences using technology. The results showed that students had positive perceptions in terms of continuance intention, satisfaction, and confirmation. That is, the participants were willing to continue their technology-assisted learning and they were satisfied with it. Our findings are similar to previous studies (Huang et al., 2017; Shadiev et al., 2021a,b). In the study of Shadiev et al. (2021b), the students had a positive attitude toward the learning activities supported by 360-degree technology, were satisfied with the technology, and had intentions to use it in the future for learning. Shadiev et al. (2021a) also reported that the participants accepted 360-degree VR technology in terms of its usefulness for cross-cultural learning and ease of use. We need to acknowledge that we cannot directly compare our results with those in previous studies on students’ creativity, innovation, and entrepreneurship because not many researchers explored such important abilities in the context of cross-cultural learning.

Our results suggest that students’ creativity, innovation, and entrepreneurship were significantly enhanced after participating in learning activities. The following reasons may explain such benefits. Firstly, in this study, peer communication was designed, including pair/group and square discussion. Students can show their individual differences such as cognitive tendency, thinking style, and individual values. Through peer communication. Therefore, communication promoted the collision of sparks of thinking among students, let students learn from each other’s strengths and weaknesses, helped students gain the cultural knowledge of different nationalities, and then improved students’ creativity, innovation, and entrepreneurship in learning activities (Ann Bainbridge Frymier, 2005; Holliday et al., 2021).

Secondly, we also found an interesting phenomenon, in the cultivation of such abilities as creativity, innovation, and entrepreneurship, the existence of a facilitator was important and necessary. In this study, facilitators were onlookers. After communicating with peers, facilitators intervened in a timely manner to guide students to comprehensively consider and modify their content. The role of a facilitator was to help students improve their content, discuss ideas how to improve content or facilitator could point out issues that students failed to notice (Goodyear and Dudley, 2015). Therefore, the help of the facilitator in this learning project was beneficial in improvement of students’ innovation, creativity, and entrepreneurship.

Thirdly, students conducted self-reflection after communicating with peers and onlookers. Students evaluated their content, thought about feedback such as comments and suggestions from peers and onlookers, and then modified content to improve its cultural, creative, innovative, and entrepreneurial value (Bower et al., 2011).

Fourthly, the cultural topics and locations to create panoramic tours were selected by students themselves. Therefore, the learning process was meaningful to students and relevant to their daily life situations. This could stimulate students’ interest and make their learning process not so difficult or challenging (Huang et al., 2017; Lin et al., 2020; Shadiev et al., 2022b). At the same time, the participants signed up voluntarily, they collaborated with international partners, and used new technologies; all of these could highly motivate their participation (Shadiev et al., 2021b). They developed a certain novelty and curiosity about the whole learning activity. Therefore, the students were very actively involved in the entire study, and their innovation, creativity, and entrepreneurship were enhanced.

Finally, technology used in the study was easy to use and useful for learning. After selecting their preferable cultural topics, students could create panoramic tours that showed cultural location, objects and people there with 360-degree view. In addition, students could experience foreign culture more contextually, immersively, and intuitively through VR panoramic tours. Therefore, students had positive perceptions of their experiences with technology (Huang et al., 2017; Lin et al., 2020; Shadiev et al., 2022a).

## Conclusion

To the best of our knowledge, most studies on cross-cultural learning supported by technologies focused on improving participants’ cross-cultural competencies such as knowledge or interaction skills. Little attention was paid to development of high-order skills such as creativity, innovation, and entrepreneurship. This study aimed to address this research gap. To this end, learning activities supported by 360-degree video technology were developed with focus on students’ creativity, innovation, and entrepreneurship.

Our results showed that learning activities were beneficial for creativity, innovation, and entrepreneurship, and students had positive perceptions of their learning experiences. Based on our results, we make the following two suggestions. First, we recommend using panoramic tour based on 360-degree video technology in the learning process. It can create authentic, contextual cross-cultural learning environments. Such environments are virtual, immersive and give good sense of presence. One of the advantages is that technology was easy to use. Students can take panoramic photographs according to their ideas and then create cross-cultural panoramic works, which is conducive to students’ full use of their subjective initiative and enthusiasm to participate in cross-cultural learning activities. At the same time, the advantage of 360-degree video technology is that it can provide students with immersion to their learning experiences and a high sense of presence. It can completely restore the virtual environment in reality, allowing students to be immersed in the scene and better experience foreign cultures. Therefore, students participating in our learning activities had an enjoyable experience. Moreover, this study designed a telecollaboration platform, where students could freely communicate with their peers and constantly modify and improve their content, providing a novel and convenient way for learning.

Second, it is suggested to have representatives of different cultures in cross-cultural learning activities. Representatives from two cultures participated in this research. The technology used in the study created learning environments in which the participants were able to exchange authentic information related to culture in creative, innovative, and entrepreneurial way, interact with each other, and experience authentic culture of their partners from different culture. Such design can be beneficial for cross-cultural learning as well as for developing such skills as creativity, innovation, and entrepreneurship.

Third, we suggest that the development of students’ abilities such as creativity, innovation, and entrepreneurship in cross-cultural learning activities supported by technology needs to be further explored in the future. We found that our learning activities effectively facilitated students’ creativity, innovation, and entrepreneurship. However, related studies paid little attention to this aspect. For example, future studies may explore such factors as peer communication, guidance of the instructor, and student self-reflection in learning activities, and how helpful each of them can be in enhancing participants’ creativity, innovation, and entrepreneurship.

## Limitation and Future Directions

In this study, the sample size of participants was small. Therefore, generalizing the results to wider population can be problematic. Future studies may consider this limitation and involve more participants. Although, this study collected and triangulated the data from different data sources to make our findings more nuanced and robust, we did not involve a control group. For this reason, we do not have any experimental evidence of the effectiveness of our intervention on creativity, innovation, and entrepreneurship. Future studies may consider testing the effectiveness of intervention in the experiment by involving control and experimental groups and comparing their learning outcomes.

In the future study, we also look forward to exploring more students’ higher-order thinking abilities, such as critical thinking, synthesis and evaluation of cultural learning content in a technology-assisted learning environment. From this perspective, we can explore how technology-supported cross-cultural learning affects these higher-order thinking abilities more specifically. Additionally, we discussed students’ creativity, innovation, and entrepreneurship from an individual perspective in this study. In the future, we plan to design teamwork sessions to investigate whether teamwork affects students’ creativity, innovation, and entrepreneurship in cross-cultural panoramic VR tours.

## Data Availability Statement

The raw data supporting the conclusions of this article will be made available by the authors, without undue reservation.

## Ethics Statement

The studies involving human participants were reviewed and approved by the School of Education Science, Nanjing Normal University. Written informed consent to participate in this study was provided by the participants’ legal guardian/next of kin.

## Author Contributions

RS, SY, and CD contributed to the conception and design of the study. SY, CD, and WS carried out the learning activity, collected the data, and analyzed the data. SY wrote the first draft of the manuscript. RS prepared the final version of the manuscript. All authors contributed to the article and approved the submitted version.

## Conflict of Interest

The authors declare that the research was conducted in the absence of any commercial or financial relationships that could be construed as a potential conflict of interest.

## Publisher’s Note

All claims expressed in this article are solely those of the authors and do not necessarily represent those of their affiliated organizations, or those of the publisher, the editors and the reviewers. Any product that may be evaluated in this article, or claim that may be made by its manufacturer, is not guaranteed or endorsed by the publisher.
